# Clinical impact of variability on CT radiomics and suggestions for suitable feature selection: a focus on lung cancer

**DOI:** 10.1186/s40644-019-0239-z

**Published:** 2019-07-26

**Authors:** Seung-Hak Lee, Hwan-ho Cho, Ho Yun Lee, Hyunjin Park

**Affiliations:** 1 Departement of Electrical and Computer Engineering, Sungkyunkwan University, Suwon, 16419 South Korea; 2 Center for Neuroscience Imaging Research, Institute for Basic Science (IBS), Suwon, 16419 South Korea; 3 Department of Radiology, Samsung Medical Center, Sungkyunkwan University School of Medicine, 81 Irwon-Ro, Gangnam-Gu, Seoul, 06351 South Korea; 4 Department of Health Sciences and Technology, SAIHST, Sungkyunkwan University, Seoul, 06351 South Korea; 5 School of Electronic and Electrical Engineering, Sungkyunkwan University, Suwon, 16419 South Korea

**Keywords:** Radiomics, Computed tomography, Feature reproducibility, Guideline for multi-center analysis, Precision medicine

## Abstract

**Background:**

Radiomics suffers from feature reproducibility. We studied the variability of radiomics features and the relationship of radiomics features with tumor size and shape to determine guidelines for optimal radiomics study.

**Methods:**

We dealt with 260 lung nodules (180 for training, 80 for testing) limited to 2 cm or less. We quantified how voxel geometry (isotropic/anisotropic) and the number of histogram bins, factors commonly adjusted in multi-center studies, affect reproducibility. First, features showing high reproducibility between the original and isotropic transformed voxel settings were identified. Second, features showing high reproducibility in various binning settings were identified. Two hundred fifty-two features were computed and features with high intra-correlation coefficient were selected. Features that explained nodule status (benign/malignant) were retained using the least absolute shrinkage selector operator. Common features among different settings were identified, and the final features showing high reproducibility correlated with nodule status were identified. The identified features were used for the random forest classifier to validate the effectiveness of the features. The properties of the uncalculated feature were inspected to suggest a tentative guideline for radiomics studies.

**Results:**

Nine features showing high reproducibility for both the original and isotropic voxel settings were selected and used to classify nodule status (AUC 0.659–0.697). Five features showing high reproducibility among different binning settings were selected and used in classification (AUC 0.729–0.748). Some texture features are likely to be successfully computed if a nodule was larger than 1000 mm^3^.

**Conclusions:**

Features showing high reproducibility among different settings correlated with nodule status were identified.

**Electronic supplementary material:**

The online version of this article (10.1186/s40644-019-0239-z) contains supplementary material, which is available to authorized users.

## Background

Precision medicine is an important factor in modern oncology, and medical imaging plays an important role [1, 2]. Radiologists have extracted clinically meaningful information related to screening, diagnosis, and treatment planning for various cancer types. Traditionally, a few imaging features specific to disease have been computed [3, 4]. For example, tumor size is the most widely used feature to asses treatment response [5, 6].

Recently, radiomics has been widely accepted in precision medicine [7]. Radiomics is an emerging research field to extract mineable high-dimensional data from clinical images [8–10]. Radiomics can be applied to various disease types with minor modifications because the feature set is likely to include effective features that cover a broad range of disease types. The results of radiomics might complement the information of tissue sampling and circulating biomarkers [11, 12].

The radiomics has shortcomings. One major shortcoming is the low reproducibility of radiomics features, which makes it difficult to compare and interpret radiomics studies. Typically, features were defined mathematically using factors affected by imaging parameters such as voxel resolution and reconstruction methods [13, 14]. Studies have proposed standardized image settings, to improve feature stability [1]. However, such standardization approaches are not always feasible for multi-center retrospective studies that might involve heterogeneous image settings. This study focused on voxel geometry (i.e., isotropic vs. anisotropic) and the number of histogram bins among the many factors affecting feature stability. A given region of interest (ROI) is made of many voxels, and voxel geometry affects features. Many features depend on the histogram of intensity from the ROI, and thus how histograms are binned affects features [15].

There are many categories within radiomics features, such as histogram-based features and texture-based features. The features may be unstable depending on the factors described above. Furthermore, some features might fail to be computed. For example, a very small nodule cannot be used to compute texture features. Inspecting the physical properties of failed computations might lead to valuable insights into performing radiomics studies.

Here, we aimed to find features showing high reproducibility with respect to voxel geometry and the number of bins for lung nodules smaller than 2 cm tested on two different cohorts (n1 = 180 and n2 = 80) by lung CT. Smaller nodules were chosen because larger nodules are likely to have less variability [16]. As a secondary aim, we tried to provide guidelines for computing features by inspecting the physical properties of failed radiomics computations.

## Material and methods

### Patients

Institutional review board (IRB) approvals from Samsung Medical Center (SMC) and Sungkyunkwan University were obtained for this retrospective study with waivers of informed consent. Two independent cohorts were employed: For the training cohort (local data), we used 180 CT images (benign: 51 and malignant: 129) from 114 patients. The nodules were less than 2 cm. Some patients (*n* = 66) had nodules defined in two time points and others had nodules defined in single time point. All the malignant nodules were confirmed as adenocarcinoma histologically in the training cohort. The benign nodules were not confirmed invasively. Using CT imaging observations, we classified nodules as benign if they showed no change for 2 years or more for the solid lesion. For sub-solid nodules, the interval was 3 years or more. For the test cohort (public data), 80 CT images from the lung nodule analysis (LUNA) database (benign: 30 and malignant: 50) were randomly chosen [17, 18]. The training cohort was used to identify reproducible features and the testing cohort was used to see if the finding generalizes to an independent data.

### CT imaging

CT images of the training set were obtained with the following parameters: detector collimation was 1.25 or 0.625 mm, the tube peak potential energies ranged from 80 to 140 kVp, tube current ranged from 150 to 200 mA, and reconstruction interval ranged from 1 to 2.5 mm. All CT images were displayed at standard mediastinal (window width, 400 HU; window level, 20 HU) and lung (window width, 1500 HU; window level, − 700 HU) window settings. In-plane resolution varied from 0.49 to 0.88 with a mean and standard deviation (SD) of 0.7 and 0.07, respectively. The mean slice thickness of images was 2.33 (range: 1-5 mm) and the SD was 0.98.

CT images of the test set were obtained from various institutions. Full details of imaging parameters are available [18]. The tube peak potential energies ranged from 120 kV to 140 kV, tube current ranged from 40 to 627 mA, the mean effective tube current was 222.1 mAs, and the reconstruction interval ranged from 0.45 to 5.0 mm. In-plane resolution varied from 0.49 to 0.9 with a mean and SD of 0.66 and 0.08, respectively. The mean value of slice thickness was 1.86 (range: 0.625–2.5 mm) and the SD was 0.52. All CT images of both cohorts were reconstructed using the standard algorithm.

### Nodule segmentation and pre-processing

On axial CT images, nodules were segmented using in-house semi-automated software by single expert [19]. Target regions were defined as nodules less than 2 cm.

For the first experiment, features computed using default voxel and isotropic voxel settings were compared. The default setting refers to native voxels (can be non-square) and the isotropic voxel setting refers to resampling imaging data into square voxels. Such a resampled square voxel setting is necessary for the following reasons. Different voxel sizes must be compared in multi-center studies, a process that usually involves reformatting imaging data into a larger voxel setting. It is undesirable to up-sample large voxels to small voxels because the process potentially involves interpolation with bias. It is preferable to down-sample small voxels to large voxels, and thus simple averaging occurs during the process. Radiomics studies evaluate texture features that require directional voxel neighborhood information. Square voxel settings are ideal because in-plane and out-of-plane directions have the same spatial sampling. The imaging data were resampled to 2x2x2 mm^3^ isotropic voxel settings using the ANTs software [20]. We were comparing data obtained from different settings and it was safe to resample to a poor resolution for a fair comparison. The training cohort had an average slice thickness of 2.33 mm, while the test cohort had an average slice thickness of 1.86 mm. Thus, we chose 2 mm as the slice thickness and made the voxel geometry isotropic to compute texture features in a standard manner.

### Experiment 1 (original vs. isotropic voxels)

A total of 252 features were considered for each voxel setting using a combination of open source code (i.e., PyRadiomics) and in-house code implemented in MATLAB **(**MathWorks, Inc.) [21]. Some of the features could not be computed and we only analyzed 128 features out of the 252 features. Further details regarding the computation failures are given in later sections.

The features were divided into four categories. Histogram-based features were calculated from four types of ROIs: whole ROI (number of features = 19), positive voxel of the whole ROI (*n* = 14), outer 1/3 of the whole ROI volume (outer ROI, *n* = 9), inner 2/3 of the whole ROI volume (inner ROI, n = 9), and the difference between outer and inner ROI (ROI delta, n = 9) [22, 23]. A given ROI was partitioned into inner and outer ROIs purely based on the volume using binary morphological operations.

A total of ten 3D shape features were calculated, and some shape features (*n* = 3) were computed from 2D data obtained from the slice where the nodule was the largest. Shape features related to nodule margin were computed using the sigmoid function (*n* = 6) [24]. The sigmoid function was used to fit density change along a sampling line drawn orthogonal to the nodule surface. Each sampling line going through one voxel on the tumor surface has a certain length (3, 5, and 7 mm in this work) inside and outside the nodule. The fractal dimension was calculated as a fractal-based feature using the box-counting method and fractal signature dissimilarity (FSD) was calculated using the blanket method [25, 26]. The lacunarity was also calculated to assess the texture or distribution of the gap.

Texture features were calculated using a Gray-level co-occurrence matrix (GLCM), intensity size zone matrix (ISZM), and neighborhood gray tone difference matrix (NGTDM) with 3D ROI [27–29]. Two types of 3D GLCM features were computed: GLCM of the whole ROI and GLCM using sub-sampled ROI. Each type was applied to four ROI types: whole, inner, outer, and delta ROIs. Intensities were binned with 256 bins. Total of 44 GLCM features were eventually obtained. Two ISZM features were computed. A 32 × 256 matrix was constructed in which the first dimension is binned intensity and the second dimension is the size. The ISZM features can quantify how many sub-regions there are and how often certain sub-regions occur within the ROI. Two features were calculated using ISZM. NTGDM-based features (*n* = 5) quantify the difference between a gray value and the average gray value of its neighbors.

Filter-based features (*n* = 9) were considered. The 3D Laplacian of Gaussian (LoG) filter was adopted [30]. Sigma values of the LoG filter were computed with σ = 0.5–3.5 in 0.5 voxel increments. Computed features were normalized to the z-score***.*** Full details of all features are given in the Additional file 1.

**Fig. 1:**
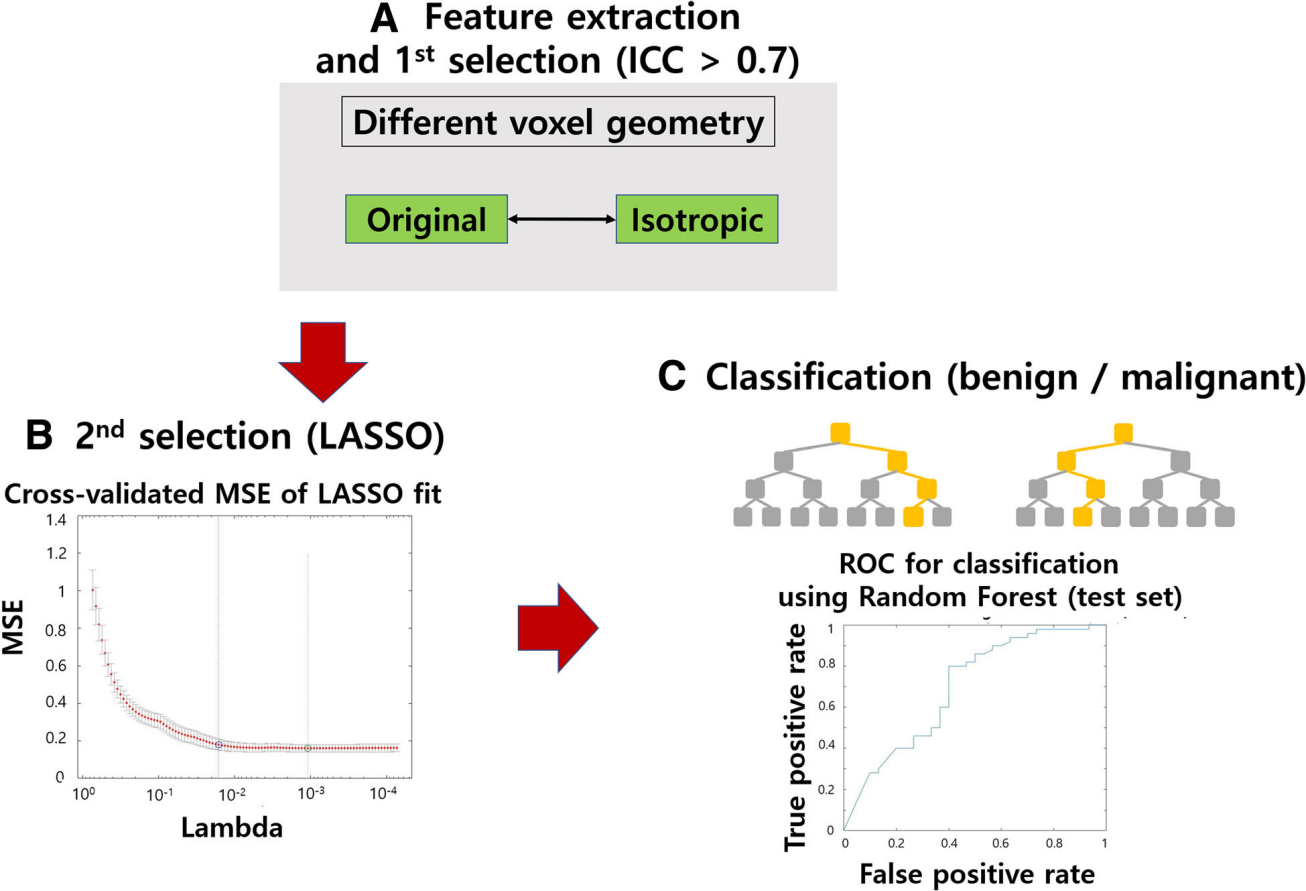
Overall design for Experiment 1. **a** Feature extraction and the 1st selection step. In the 1st selection step, we selected features with ICC **≥** 0.7. **b** In the 2nd selection, we applied LASSO to select features that can explain nodule status. **c** The features were used to train a RF classifier to classify nodule status. It was later tested in a test cohort

Features with high reproducibility were identified as those with intra-class correlation (ICC) over 0.7 between two voxel settings (original vs. isotropic) using SPSS (IBM Corp.) [31]. The least absolute shrinkage selector operator (LASSO) was used to select features to explain nodule status (i.e., malignant vs. benign) for each voxel setting [32, 33]. The features common to both settings were retained. Thus, features that were both reproducible and correlated with nodule status were identified. The effectiveness of the identified features was further assessed by using the features to classify between malignant and benign nodules in both the training and testing sets. The overall design of experiment 1 is in Fig. 1.

### *Experiment 2* (default bin setting vs. changed bin setting)

Many radiomics features are computed from 1D or 2D histograms. In our study, histogram-, GLCM-, and ISZM-based features depend on histograms. The histograms are dependent on the number of bins adopted. The default number of bins was compared with other numbers of bins. There were 4096 bins as the default setting for histogram-based features accounting for the CT intensity range [31]. The default bins were 256 for GLCM and 32 for ISZM. For histogram-based features, the default bin (4096 bin) setting was compared using 256, 512, 1024, and 2048 bins. For GLCM-based features, default bin setting (256 bin) was compared with those using 32, 64, and 128 bins. For ISZM-based features, default bin setting (32 bin) were compared with those using 16 and 64 bins. The histogram-, GLCM-, and ISZM-based features were computed as described in the first experiment.

**Fig. 2:**
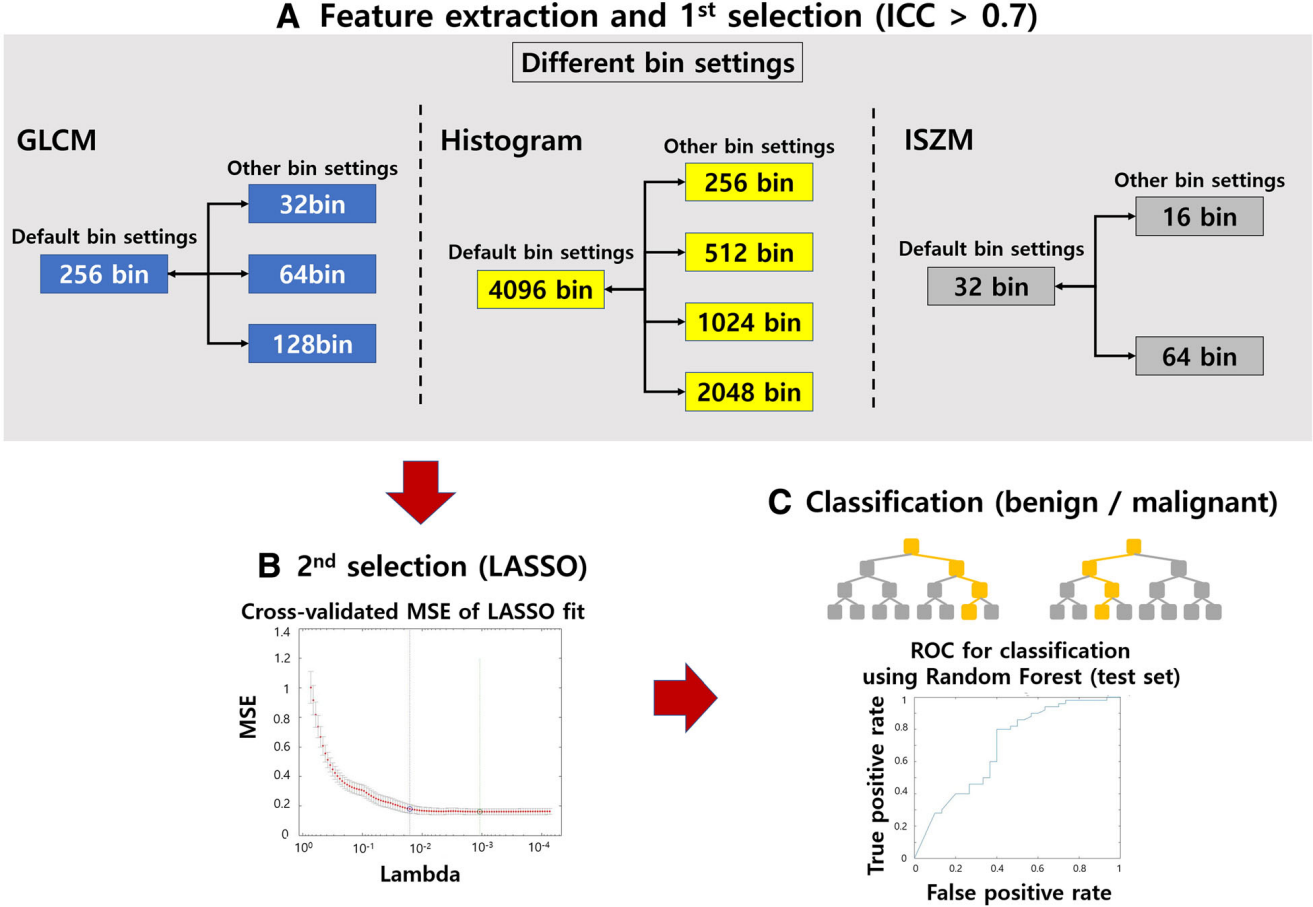
Overall design for Experiment 2. **a** Feature extraction and the 1st selection step. In the 1st selection step, we selected features with ICC **≥** 0.7. In this process, we found that both histogram- and ISZM-based features have ICC **≥** 0.9. Thus, we fixed the histogram- and ISZM-based features to the default bin settings. **b** In the 2nd selection, we applied LASSO to select features that can explain nodule status. **c** The features were used to train a RF classifier to classify nodule status. It was later tested in a test cohort

The ICC between features from different bin settings (default vs. changed bin settings) was calculated to identify features showing high reproducibility. Features with ICC values higher than 0.7 were retained [31]. The LASSO was then applied to select features that can explain nodule status (i.e., malignant vs. benign) for each binning setting. Common features from the compared settings were retained and used for classification of nodule status. The overall design of experiment 2 is in Fig. 2.

### Inspection of failed computation for features

Some features failed to be computed in the extraction step. The following features were excluded because of high error rate: histogram-based features (positive pixel, inner ROI, outer ROI, and delta ROI features), GLCM features (inner ROI, outer ROI, and delta ROI), sub-sampled GLCM features, and NGTDM features. These features were not computed because nodules in this study were too small.

The physical properties of failed computation cases (error group) and successful computation cases (non-error group) were compared for the two feature categories using one-tailed t-tests. Since all cases had histogram- and shape-based features available, those features were used to compare the two groups. In addition, the histogram/shape-based features are easily interpretable which makes them good features to compare the two groups. A total of 26 features (19 histogram-based features and 7 shape-based features) were compared between the two groups.

### Statistical analysis

The features identified from the two experiments were used as inputs for random forest (RF) classifier to distinguish between malignant and benign nodules [34]. The RF classifier used 200 decision trees. The classifier was trained using data of the training set, and it was then applied to the test set. The area under the curve (AUC), sensitivity, specificity, and accuracy of the receiver operating characteristic (ROC) curve were measured. All statistical analysis procedures were calculated using MATLAB.

## Results

### Experiment 1 (original vs. isotropic)

**Fig. 3:**
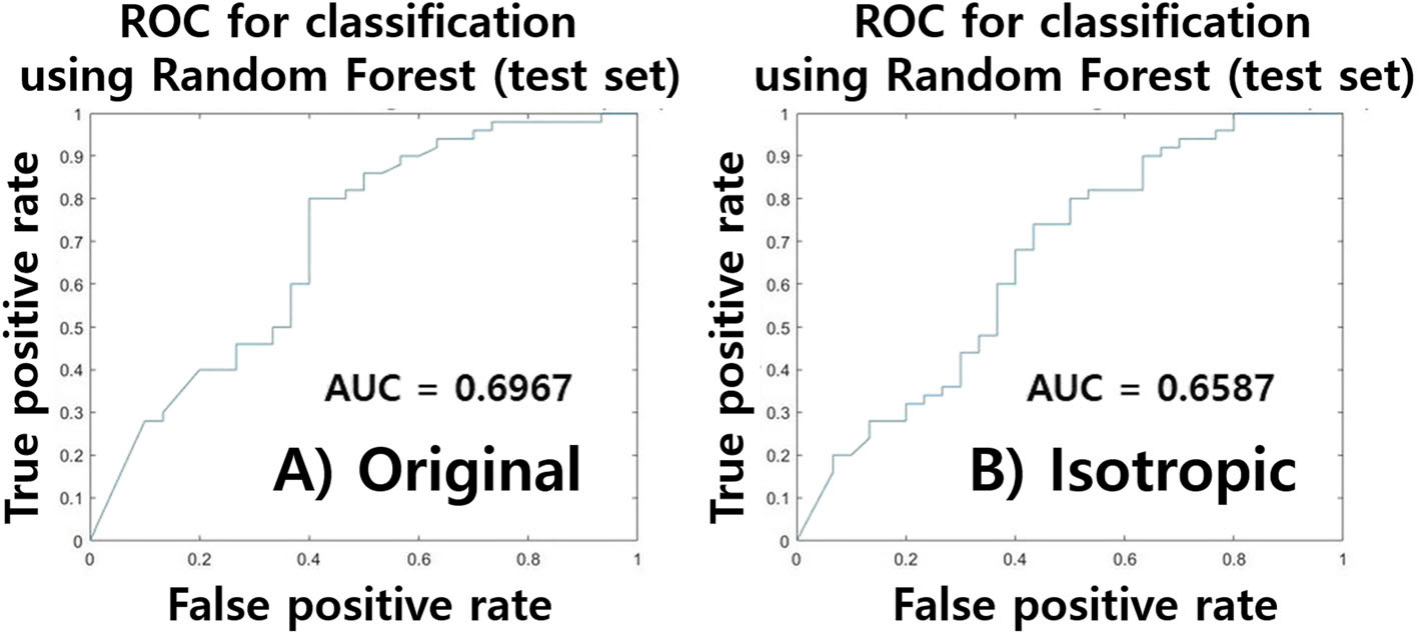
Performance curve of the RF classifier in the test set. **a** shows the receiver operating characteristic (ROC) curve of the original voxel setting and **b**) shows the ROC curve of the isotropic voxel setting

From the training data, features computed using default voxel and isotropic voxel settings were compared. Thirty-eight features (ICC > 0.7) were selected from 252 features. Of these, 23 features (13 for the original voxel and 10 for isotropic voxel settings) that can explain nodule statues (malignant/benign) were retained using LASSO. Nine features were common between the two voxel settings: maximum, minimum (histogram-based), maximum 3d diameter, spherical disproportion (shape-based), cluster tendency, dissimilarity, entropy (GLCM), skewness_1 (LoG filter-based), and lacunarity (fractal-based). Skewness_1 refers to the skewness of intensity within the ROI filtered using the LoG filter with σ =1. These features are reproducible and correlated with nodule status with respect to two voxel settings. The selected features were referred to as signatures. The features were used to train a RF classifier in the training data. The RF classifier was used to classify nodule status (benign/malignant) in the test set. The performance of the classification is shown in Table 1. Associated AUC plots are shown in Fig. 3. We quantified how each identified radiomics feature contributed to explaining the nodule status and the relative importance of the features using a permutation of out-of-bag (OOB) observations within the RF classifier framework. These additional results are given in the Additional file 1.

**Table 1:** Classification performance of test set using RF for two voxel settings (Experiment 1)

	Original voxel setting	Isotropic voxel setting
Area under curve	0.6967	0.6587
Accuracy	0.7250	0.7000
Sensitivity	0.9000	0.9000
Specificity	0.4333	0.3667

### *Experiment 2* (default vs. changed bin settings)

**Fig. 4:**
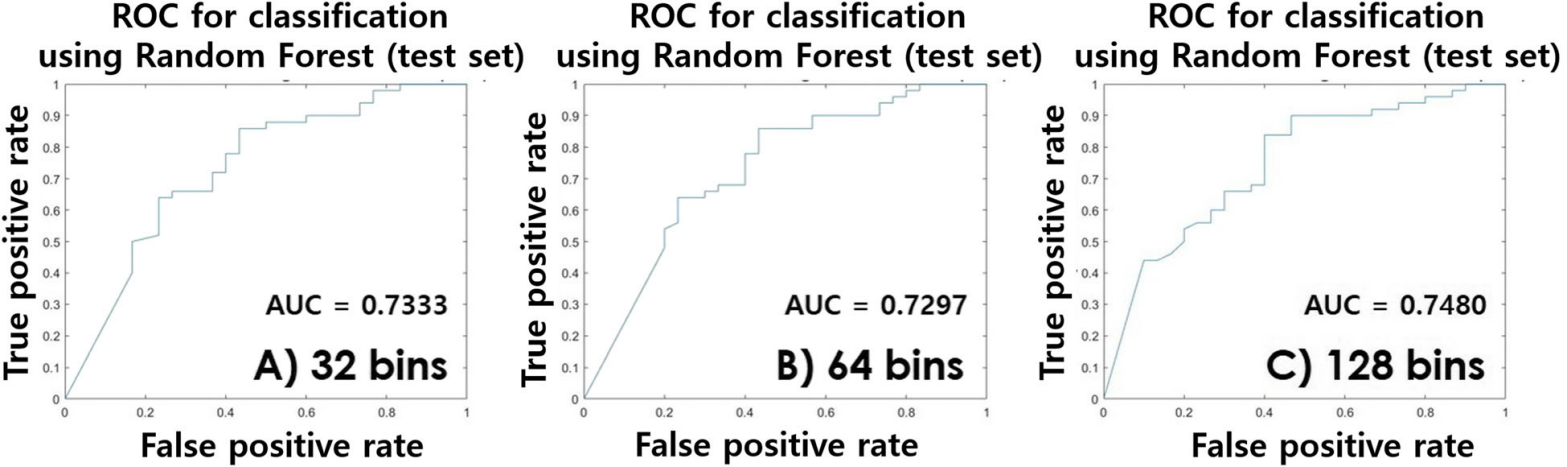
Performance curve of the RF classifier in the test set. **a** shows the receiver operating characteristic (ROC) curve of the 32 bins setting, **b**) shows the ROC curve of the 64 bins setting, and **c**) shows the ROC curve of the 128 bins setting

From training data, features computed using the default number of bins were compared with features computed using other numbers of bins. Histogram-, GLCM-, and ISZM-based features are affected by bin settings. All histogram-based features had ICC over 0.7 when comparing the default bin (= 4096) and changed settings (256, 512, 1024, and 2048). All ISZM-based features had ICC over 0.7 when comparing between the default bin (= 32) and changed settings (16 or 64). GLCM-based features showed variability. Two features (difference entropy and homogeneity) showed ICC over 0.7 between the default bin (= 256) and changed bin (= 32) settings. Twenty-four features showed high reproducibility (17 histogram-, 2 ISZM-, and 2 GLCM-based features) for the first ICC comparison. Five features (32 bins) were retained using LASSO. Three features (difference entropy, homogeneity, and informational measure of correlation [IMC]) showed ICC over 0.7 between the default bin (= 256) and changed bin (=64) settings. Twenty-five features showed high reproducibility (17 histogram-, 2 ISZM-, and 3 GLCM-based features) for the second ICC comparison. Six features (64 bins) were retained using LASSO. Comparison of the default bin (= 256) and changed bin (= 128) settings showed that maximum probability, difference entropy, dissimilarity, energy, entropy, homogeneity, and IMC had ICC over 0.7. There were 29 (17 histogram-, 2 ISZM-, and 7 GLCM-based features) features showing high reproducibility for the third ICC comparison. Six features (128 bins) were retained using LASSO. The maximum, minimum, entropy (histogram-based), difference entropy, and homogeneity (GLCM) features were common between three settings (16, 64, and 128 bins). The common features of the three settings are reproducible and correlated with nodule status with respect to the different GLCM binning settings. These common features were referred to as signatures. The features were used to train a RF classifier in the training data. The RF classifier was used to classify nodule status (benign/malignant) in the test set. The performance of the classification is given in Table 2. Associated AUC plots are shown in Fig. 4. Table 3 reports features showing high reproducibility from two experiments and their possible interpretations. As in experiment 1, the results for contribution of radiomics features are given in the Additional file 1.

**Table 2:** Classification performance of test set using RF for different GLMC bin settings (Experiment 2)

	32 bins	64 bins	128 bins
Area under curve	0.7333	0.7297	0.7480
Accuracy	0.7250	0.7250	0.7375
Sensitivity	0.8800	0.8600	0.9000
Specificity	0.4667	0.5000	0.4667

**Table 3:** Features showing high reproducibility from two experiments

	Category	Parameter	Description / Interpretation
Experiment 1	Histogram-based features	Maximum	Measures maximum intensity value of histogram
		Minimum	Measures minimum intensity value of histogram
	Shape-based features	Maximum 3d diameter	Measures maximum 3D ROI diameter as the largest pairwise Euclidean distance between surface voxels of the ROI
		Spherical disproportion	Ratio of the surface area of the ROI to the surface area of a sphere with the same volume as the ROI
	Texture-based features (GLCM)	Custer tendency	Measures homogeneity of GLCM
		Dissimilarity	Measures differences of entries in GLCM
		Entropy	Measures irregularity of GLCM
	Filter-based feature	Log Skewness (*σ* = 1)	Measurement of skewness of ROI image processed by log filter
	Fractal-based feature	Lacunarity	Measure of the texture or distribution of gaps within an image
Experiment 2	Histogram-based features	Maximum	Same as experiment 1
		Minimum	Same as experiment 1
		Entropy	Measures irregularity of histogram
	Texture-based features	Difference entropy	Measures entropy of processed GLCM matrix Px-y
		Homogeneity	Measures closeness of GLCM

### Suggested guidelines from inspecting failed computation cases

**Fig. 5:**
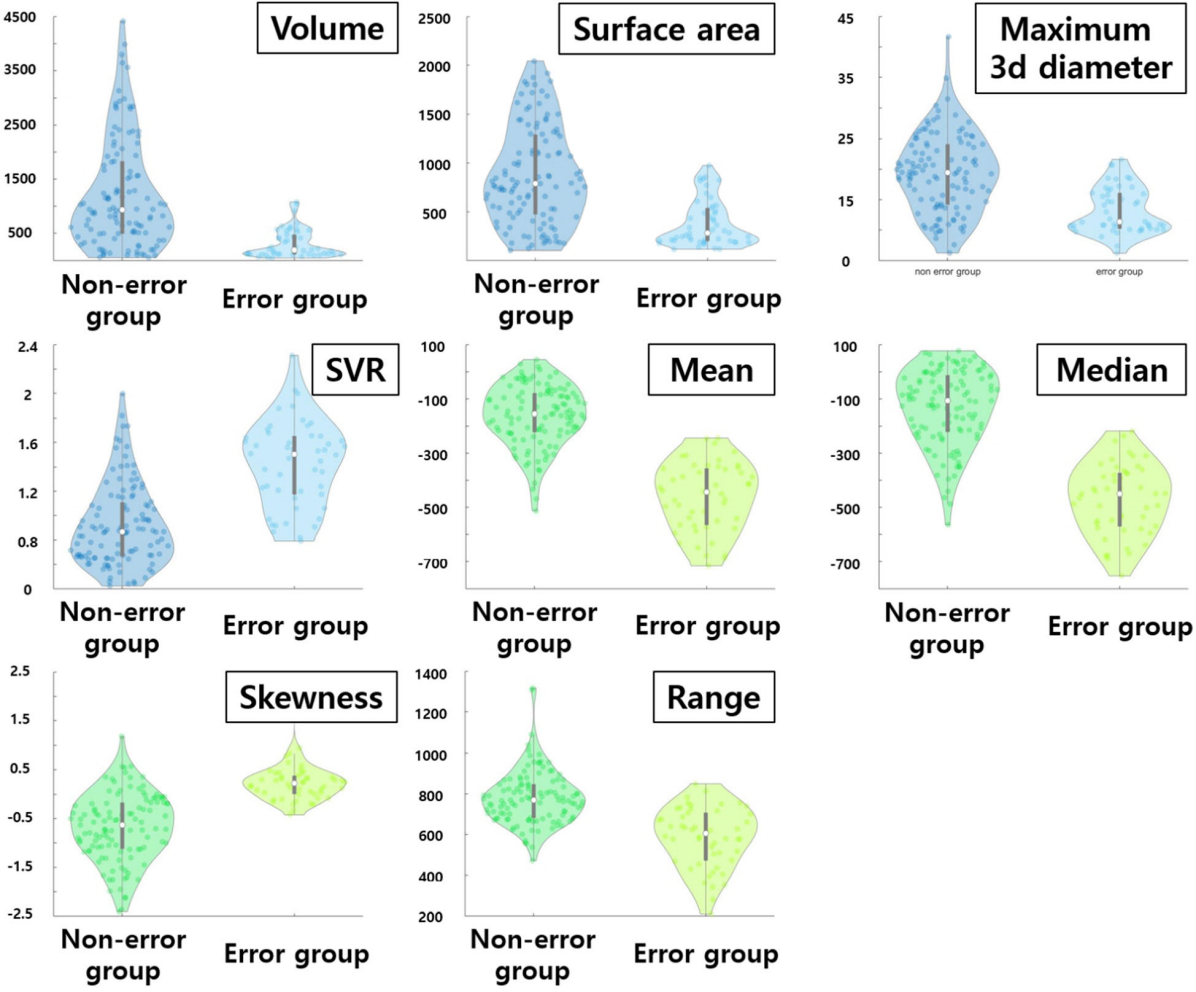
Various features compared between the error and non-error groups related to computation of NGTDM features. Blue plots were the difference between shape-based features, and green plots were differences between histogram-based features

The properties of cases with failed NGTDM computation using histogram- and shape-based features were further examined. One notable difference was from the skewness of histogram-based features. The skewness of the error group (mean 0.24) was larger than that of the non-error group (mean − 0.67). This indicates that the non-error group tends to have higher mean intensities. The volume of the non-error group (mean 1228.89 mm^3^) was larger than that of the error group (mean 470.30 mm^3^). The 95% confidence interval (CI) of volume features for the non-error group is 1045.5mm^3^ to 1412.28mm^3^. The CIs for various features that differed between the error and non-error groups are reported in Table 4. Figure 5 shows various features compared between error and non-error groups. We recommend that nodules should be larger than a certain size (≥ 1000 mm^3^) and the intensity values should be brighter than the average intensity of the nodule for successful computation of NGTDM features.

**Table 4:** Confidence interval of various features for non-error group related to the failure of NGTDM

Shape feature	Volume	Maximum 3d diameter	Surface area	Surface volume ratio
	1045.5 ~ 1412.28	18.15 ~ 20.46	780.5 ~ 964.07	0.86 ~ 0.98
Histogram feature	Mean	Skewness	Range	Median
	− 182.03 ~ −141.26	−0.8 ~ −0.55	756.35 ~ 805.08	− 158.52 ~ − 107.86

**Fig. 6:**
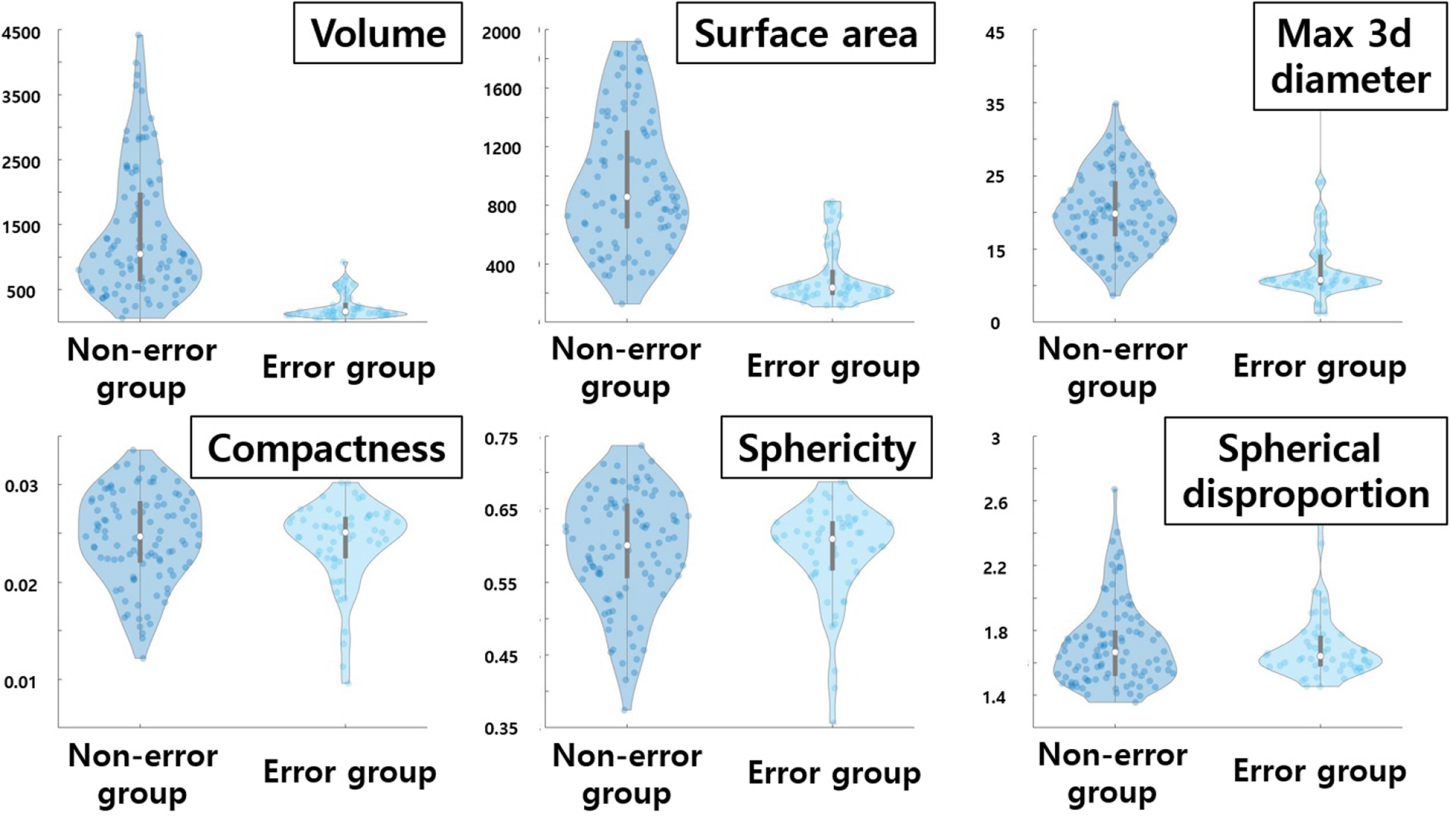
Various features compared between error and non-error groups related to computation of sub-sampled GLCM features. Blue plot on the right is for the non-error group and light blue plot on the left is for the error group

The properties of cases with failed sub-sampled GLCM computation were also examined. The volume related features (volume, surface area, and maximum 3D diameter) of the non-error group were larger than those of the error group. However, compactness, sphericity, and spherical disproportion values, which are independent of size, did not differ between the two groups. CIs were applied to calculate the range of features to set recommended criteria for which sub-sampled GLCM features can be computed. According to the calculated values, sub-sampling GLCM features can be calculated when the volume is 1100 mm^3^ or more, maximum 3d diameter value is 19 mm or more, and surface area value is 870 mm^2^ or more. The comparison plot between groups and confidence interval values are shown in Fig. 6 and Table 5, respectively.

**Table 5:** Confidence interval of various features for non-error group related to the failure of sub-sampled GLCM

Shape feature	Volume	Maximum 3d diameter	Surface area
	1186.17 ~ 1567.5	19.37 ~ 21.34	871.56 ~ 1045.96
	Compactness	Sphericity	Spherical disproportion
	0.024 ~ 0.025	0.58 ~ 0.61	1.66 ~ 1.76

## Discussion

Our goal was not to find features that lead to a good classification of nodule status but to find reproducible features between different settings (voxel geometry and binning settings). We observed that the classification performance using the reproducible features stayed similar, which could be indirect evidence of reproducibility of the identified features. We identified nine features showing high reproducibility that correlate with nodule status regardless of voxel geometry settings (isotropic vs. anisotropic). We also identified six features showing high reproducibility correlated with nodule status regardless of binning settings.

There are 35 papers related to reproducibility of radiomics between 2010 and 2017 according to a review article [35]. Existing studies on average used 62 samples in the training cohort, while ours used 114 samples in the training cohort, which would lead to better statistical robustness. Many studies lacked independent test cohorts, while we validated the reproducible features in an independent test cohort [36, 37]. The existing studies reported divergent sets of reproducible features. This is rather expected because the training cohort varied significantly among studies.

The training cohort included only small (< 2 cm) nodules. The randomly chosen test cohort from the LUNA database was confirmed to be small. The maximum 3D diameter of the test cohort was on average 2.1 cm, while that of the training cohort was 1.6 cm. There is a scarcity in studies dealing with reproducibility in lung radiomics, especially for small nodules. Our study tried to fill that gap in research.

There are limited CT imaging studies focusing on small lung nodules. One radiomics study reported 84% accuracy in distinguishing between benign and malignant cases in small nodules [38]. Another radiomics study reported AUC of 0.80 using a RF classifier [39]. The first two studies considered different sets of radiomics features including Laws and margin sharpness features and thus the features identified from them could not be compared directly with the identified features of our study. Mehta et al. used the volume of the nodules to distinguish between benign and malignant nodules and reported similar AUC compared to ours [40]. All these studies lacked validation using independent cohorts and thus the performance values could be inflated. In addition, our study did not try to find radiomics features that led to good classification performance but sought reproducible features between different settings (voxel geometry and binning settings). Thus, our study could have lower classification performance and lead to a different set of radiomics features compared to existing studies on small lung nodules.

We identified nine features showing high reproducibility that correlate with nodule status regardless of voxel geometry settings (isotropic vs. anisotropic): maximum, minimum (histogram-based), maximum 3d diameter, spherical disproportion (shape-based), cluster tendency, dissimilarity, entropy (GLCM), skewness_1 (LoG filter-based), and lacunarity (fractal-based). Most (= 26) of the histogram and shape-based features had ICC over 0.7, and selected features were those related to nodule status. Existing studies also identified maximum, minimum (histogram-based), maximum 3d diameter, and spherical disproportion (shape-based) as important features related to nodule status. GLCM features involve directional assessment of neighborhood voxels, which differs largely among voxel geometry settings. In the isotropic setting, directions have 45-degree increments, while in the anisotropic setting, directions have different increments. Only a few GLCM features were reproducible (ICC over 0.7), and the identified reproducible features correlated with nodule status. This is one novel finding of our study. Features of the LoG category operated on many scales denoted by sigma. Some features of the LoG category were reproducible, and those with small sigma were suitable for small nodules and could be selected (e.g., skewness σ = 1). Fractal features quantify shape in a multi-scale fashion and thus can be insensitive to voxel geometry settings.

We identified five features showing high reproducibility correlated with nodule status regardless of binning settings: maximum, minimum, entropy (histogram-based), difference entropy, and homogeneity (GLCM) features. All histogram-based features had ICC over 0.7, and the selected features were those related to nodule status. In addition to the first experiment, entropy was identified, which is frequently found in other radiomics studies related to nodule status. GLCM features varied significantly depending on bin settings, and only 2, 3, and 7 features had ICC over 0.7 when 32, 64, and 128 bins were used, respectively, compared to the default 256 bin setting. Among these features, difference entropy and homogeneity were related to nodules status. These two features quantify texture from the entire GLCM, not some parts of it, thus, they are reproducible with respect to bin settings. ISZM features were reproducible but did not reflect nodule status. One possibility was that only small nodules (≤ 2 cm) were considered, limiting the size variability portion of the ISZM.

The properties of failed NGTDM/sub-sampled GLCM computation cases were examined using histogram- and shape-based features. We found that nodules need to be larger than a certain size (e.g., over 1000 mm^3^ for NGTDM features). The texture features require voxel neighborhood structure, and thus the ROI needs to be larger than the threshold. This could be a practical lower limit on nodule size for lung radiomics. Our results were computed from image acquisition settings of varying resolution (in-plane resolution between 0.48 mm to 0.9 mm and out-of-plane resolution from 0.6 mm to 10 mm), and the lower limit could be lower in an imaging acquisition setting with smaller voxels.

Radiomics in lung cancer is different from in other oncology fields. Lung cancer resides in an environment rich with air, while other cancers primarily consist of soft tissue and reside in the interstitium [6]. Consequently, tumor progression in lung cancer is multi-factorial. In addition to the usual volume reduction, tumor progression is associated with density change from ground-glass opacity (GGO) to solid component [3, 41, 42]. Thus, radiomics in the lung should jointly consider the tumor core and surrounding air components along with textural changes in density to properly model lung cancers. Reproducibility studies in lung radiomics are largely lacking, and our study provides suggestions for future lung radiomics studies.

Our study has limitations. We did not fully test the reproducibility of all 252 features. Our study focused on small nodules which led to uncalculated features in some categories. This was further explored comparing properties of the error and non-error group. Still, future studies need to explore reproducibility of radiomics features for larger nodules. Our results were derived from two datasets, and further validations are necessary using data of different image acquisition settings. The features we identified showed high reproducibility (via ICC) reflecting nodule status (via LASSO). If a future radiomics study requires another clinical variable (e.g., therapy response), the researchers should change the LASSO portion with appropriate clinical variables as necessary. Lung nodules are imaged using other modalities such as MRI and PET in addition to CT. Reproducibility of radiomics features is an important future research topic.

## Conclusion

We identified nine features showing high reproducibility with respect to voxel geometry and five features showing high reproducibility with respect to the number of bins for lung nodules smaller than 2 cm tested on two different cohorts. We also provided guidelines for computing features by inspecting the physical properties of failed radiomics computations. The features we identified are low dimensional (< 10) and they can be easily computed as a quick pre-screening tool to determine whether a full radiomics study is worthwhile.

## Additional file

Additional file 1: Feature extraction. (DOCX 409 kb)

## Data Availability

The data and material are available through one of the corresponding authors (Dr. Ho Yun Lee).

## Abbreviations

CI: confidence interval
GGO: ground-glass opacity
GLCM: Gray-level co-occurrence matrix
ICC: Intra-class correlation
ISZM: intensity size zone matrix
LASSO: The least absolute shrinkage selector operator
LoG: Laplacian of Gaussian
NGTDM: neighborhood gray tone difference matrix
RF: random forest

## References

[1] Lambin P, Leijenaar RTH, Deist TM, Peerlings J, De Jong EEC, Van Timmeren J (2017). Radiomics: the bridge between medical imaging and personalized medicine. Nat rev Clin Oncol. Nat Publ Group.

[2] Baumann M, Krause M, Overgaard J, Debus J, Bentzen SM, Daartz J (2016). Radiation oncology in the era of precision medicine. Nat Rev Cancer Nature Publishing Group.

[3] Chong Y, Kim JH, Lee HY, Ahn YC, Lee KS, Ahn MJ (2014). Quantitative CT variables enabling response prediction in neoadjuvant therapy with EGFR-TKIs: are they different from those in neoadjuvant concurrent chemoradiotherapy?. PLoS ONE.

[4] Grove O, Berglund AE, Schabath MB, Aerts HJWL, Dekker A, Wang H (2015). Quantitative computed tomographic descriptors associate tumor shape complexity and intratumor heterogeneity with prognosis in lung adenocarcinoma. PLoS ONE.

[5] Choi H, Charnsangavej C, Faria SC, Macapinlac HA, Burgess MA, Patel SR (2007). Correlation of computed tomography and positron emission tomography in patients with metastatic gastrointestinal stromal tumor treated at a single institution with imatinib mesylate: proposal of new computed tomography response criteria. J Clin Oncol.

[6] Kang H, Lee HY, Lee KS, Kim J-H (2012). Imaging-based tumor treatment response evaluation: review of conventional, new, and emerging concepts. Korean J Radiol.

[7] Gillies RJ, Kinahan PE, Hricak H (2016). Radiomics : images are more than. Radiology..

[8] Aerts HJWL, Velazquez ER, Leijenaar RT, Parmar C, Grossmann P, Carvalho S (2014). Decoding tumour phenotype by noninvasive imaging using a quantitative radiomics approach. Nat Commun.

[9] Rizzo S, Botta F, Raimondi S, Origgi D, Fanciullo C, Morganti AG, et al. Radiomics: the facts and the challenges of image analysis. European Radiology Experimental; 2018; 10.1186/s41747-018-0068-z PMC6234198 30426318

[10] Thawani R, Mclane M, Beig N, Ghose S, Prasanna P, Velcheti V (2018). Lung Cancer Radiomics and radiogenomics in lung cancer : a review for the clinician. Lung Cancer Elsevier.

[11] Zhang B, Tian J, Dong D, Gu D, Dong Y, Zhang L (2017). Radiomics features of multiparametric MRI as novel prognostic factors in advanced nasopharyngeal carcinoma. Clin Cancer Res.

[12] Leijenaar RTH, Carvalho S, Hoebers FJP, Aerts HJWL, Van Elmpt WJC, Huang SH (2015). External validation of a prognostic CT-based radiomic signature in oropharyngeal squamous cell carcinoma. Acta Oncol (Madr).

[13] Yip SSF, Aerts HJWL (2016). Applications and limitations of radiomics. Phys Med Biol IOP Publishing.

[14] Lee G, Bak SH, Lee HY, Choi JY, Park H, Lee S-H, et al. Measurement variability in treatment response determination for non–small cell lung Cancer. J Thorac Imaging 2019;0:1. 10.1097/RTI.0000000000000390 30664063

[15] Leijenaar RTH, Nalbantov G, Carvalho S, Van Elmpt WJC, Troost EGC, Boellaard R (2015). The effect of SUV discretization in quantitative FDG-PET Radiomics: the need for standardized methodology in tumor texture analysis. Sci Rep Nature Publishing Group.

[16] Oxnard GR, Zhao B, Sima CS, Ginsberg MS, James LP, Lefkowitz RA (2019). Variability of Lung Tumor Measurements on Repeat Computed Tomography Scans Taken Within 15 Minutes. J Clin Oncol.

[17] Ali I, Hart GR, Gunabushanam G, Liang Y, Muhammad W, Nartowt B (2018). Lung nodule detection via deep reinforcement learning. Front Oncol.

[18] Armato SG, McLennan G, Bidaut L, McNitt-Gray MF, Meyer CR, Reeves AP (2011). The lung image database consortium (LIDC) and image database resource initiative (IDRI): a completed reference database of lung nodules on CT scans. Med Phys.

[19] Song SH, Park H, Lee G, Lee HY, Sohn I, Kim HS (2017). Imaging phenotyping using Radiomics to predict micropapillary pattern within lung adenocarcinoma. J Thorac Oncol Elsevier Inc.

[20] Avants B, Tustison N, Song G. Advanced normalization tools (ANTS). Insight J. 2009:1–35.

[21] Van Griethuysen JJM, Fedorov A, Parmar C, Hosny A, Aucoin N, Narayan V (2017). Computational radiomics system to decode the radiographic phenotype. Cancer Res.

[22] Lee G, Park H, Sohn I, Lee S, Song SH, Kim H, et al. Comprehensive Computed Tomography Radiomics Analysis of Lung Adenocarcinoma for Prognostication. Oncologist. 2018;theoncologist. 2017–0538. 10.1634/theoncologist.2017-0538 PMC6058328 29622699

[23] Lee SW, Park H, Lee HY, Sohn I, Lee SH, Kang J (2018). Deciphering Clinicoradiologic phenotype for thymidylate synthase expression status in patients with advanced lung adenocarcinoma using a Radiomics approach. Sci Rep Springer US.

[24] Aerts HJWL, Grossmann P, Tan Y, Oxnard GG, Rizvi N, Schwartz LH (2016). Defining a Radiomic response phenotype: a pilot study using targeted therapy in NSCLC. Sci Rep Nature Publishing Group.

[25] Lennon FE, Cianci GC, Cipriani NA, Hensing TA, Zhang HJ, Chen C-T (2015). Lung cancer—a fractal viewpoint. Nat Rev Clin Oncol. Nat Publ Group.

[26] Wang C, Subashi E, Yin FF, Chang Z (2016). Dynamic fractal signature dissimilarity analysis for therapeutic response assessment using dynamic contrast-enhanced MRI. Med Phys.

[27] Thibault G, Fertil B, Navarro C, Pereira S, Cau P, Levy N, et al. Texture indexes and Gray level size zone matrix application to cell nuclei classification. Pattern Recognit Inf Process. 2009:140–5.

[28] Niu L, Qian M, Yang W, Meng L, Xiao Y, Wong KKL (2013). Surface roughness detection of arteries via texture analysis of ultrasound images for early diagnosis of atherosclerosis. PLoS ONE.

[29] Haralick RM, Shanmugam K (1973). Textural features for image classification. IEEE Trans Syst Man Cybern.

[30] Ganeshan B, Skogen K, Pressney I, Coutroubis D, Miles K (2012). Tumour heterogeneity in oesophageal cancer assessed by CT texture analysis: Preliminary evidence of an association with tumour metabolism, stage, and survival. Clin Radiol The Royal College of Radiologists.

[31] Liu Y, Zhang Y, Cheng R, Liu S, Qu F, Yin X, et al. Radiomics analysis of apparent diffusion coefficient in cervical cancer: a preliminary study on histological grade evaluation. J Magn Reson Imaging. 2018:1–11. 10.1002/jmri.26192 29761595

[32] Liang C, Huan Y, He L, Chen X, Ma Z, Dong D (2016). The development and validation of a CT-based radiomics signature for the preoperative discrimination of stage I-II and stage III-IV colorectal cancer. Oncotarget..

[33] Huang Y, Liu Z, He L, Chen X, Pan D, Ma Z (2016). Radiomics signature : a potential biomarker for the prediction of disease-free survival in early-stage (I or II) non—small cell lung Cancer. Radiology..

[34] Breiman LEO (2001). Random forests. Mach Learn.

[35] Traverso A, Wee L, Dekker A, Gillies R (2018). Repeatability and reproducibility of Radiomic features: a systematic review. Int J Radiat Oncol Biol Phys The Authors.

[36] Koo HJ, Sung YS, Shim WH, Xu H, Choi C, Kim HR, et al. Quantitative Computed Tomography Features for Predicting Tumor Recurrence in Patients with Surgically Resected Adenocarcinoma of the Lung. PLoS ONE. 2017:1–14. 10.1371/journal.pone.0167955 PMC5221878 28068363

[37] Coroller TP, Agrawal V, Narayan V, Hou Y, Grossmann P, Lee SW (2016). Radiomic phenotype features predict pathological response in non-small cell lung cancer. Radiother Oncol Elsevier Ireland Ltd.

[38] Chen C, Chang C, Tu C, Liao W, Wu B (2018). Radiomic features analysis in computed tomography images of lung nodule classification. PLoS ONE.

[39] Felix A, Oliveira M, Machado A. Using 3D Texture and Margin Sharpness Features on Classification of Small Pulmonary Nodules. 29th SIBGRAPI Conf Graph Patterns Images. 2016. p. 394–400.

[40] Tammemagi MC, Gomez M, Nietert PJ (2010). The utility of nodule volume in the context of malignancy prediction for small. Chest The American College of Chest Physicians.

[41] Kobayashi Y, Fukui T, Ito S, Usami N, Hatooka S, Yatabe Y (2013). How long should small lung lesions of ground-glass opacity be followed?. J Thorac Oncol International Association for the Study of Lung Cancer.

[42] Staring M, Pluim JPW, De Hoop B, Klein S, Van Ginneken B, Gietema H (2009). Image subtraction facilitates assessment of volume and density change in ground-glass opacities in chest CT. Investig Radiol.

