# Supplementary material for: Clinical impact of variability on CT radiomics and suggestions for suitable feature selection: a focus on lung cancer
Source: Cancer Imaging. 2019 Jul 26;19:54. doi: 10.1186/s40644-019-0239-z (PMC6660971; doi:10.1186/s40644-019-0239-z)
Supplement: Supplementary file 1 — Feature extraction. (DOCX 409 kb) [file 40644_2019_239_MOESM1_ESM.docx]

**Additional file 1:**

**Clinical impact of variability on CT radiomics and suggestions for suitable feature selection: A focus on lung cancer**

**Feature extraction**

A total of 252 features were computed for each voxel setting using a combination of open source code (i.e., PyRadiomics) and in-house code implemented in MATLAB (**MathWorks, Inc.) [1].** Among 252 features, 173 features were computed using the PyRadiomics, while 79 features were computed using the in-house code. The features computed using the in-house code belonged to fractal-based, positive voxel-based, and sub-sampled GLCM-based, and sigmoid-based features.

Table S1. Radiomics features based on histogram, shape and size, texture, fractal, filtered, and sigmoid function.

| **Histogram features** | | **Shape and Size features** | **Texture features** | | |
| --- | --- | --- | --- | --- | --- |
| Based on whole pixel (n=19) | Based on positive pixel (n=14) | Based on 2D, 3D image (n=13) | Based on GLCM (n=11) | Based on ISZ (n=2) | Based on NGTDM (n=5) |
| Maximum* | Mean | Compactness | Auto correlation** | Size zone variance | Busyness |
| Minimum* | Standard variation | Surface area | Cluster Tendency** | Intensive variance | Coarseness |
| Median* | Variance | Convexity | Maximum probability** |  | Complexity |
| Mean* | Maximum | Sphericity | Contrast** |  | Contrast |
| Variance* | Median | Spherical disproportion | Difference entropy** |  | Strength |
| Standard variation* | Minimum | Maximum 3D diameter | Dissimilarity** |  |  |
| Energy | Inter quartile range | Surface to volume ratio | Energy** |  |  |
| Skewness* | Range | Volume | Entropy** |  |  |
| Kurtosis* | Root mean square | Density | Homogeneity** |  |  |
| Root mean square | Skewness | Mass | Informational measure of correlation** |  |  |
| Inter quartile range | Energy | Roundness factor | Variance** |  |  |
| Range | Entropy | Eccentricity |  |  |  |
| Percentile 2.5%, 25%, 50%, 75%, 97.5% | Kurtosis | Solidity |  |  |  |
| Entropy* | Uniformity |  |  |  |  |
| Uniformity | Mean value of positive pixels |  |  |  |  |
|  | Uniformity of positive pixel |  |  |  |  |
|  |  |  |  |  |  |
| **Fractal features** | | **Filtered features (LoG*******)** | | **Sigmoid function features** | |
| Based on box-counting method (n=2) | Based on blanket method (n=1) | σ = 0.5 – 3.5 (n=9, respectively) | | 3,5,7mm (n=6, respectively) | |
| Dimension | Fractal signature dissimilarity | Mean | | Amplitude mean | |
| Lacunarity |  | Max | | Amplitude standard deviation | |
|  |  | Min | | Slope mean | |
|  |  | Median | | Slope standard deviation | |
|  |  | Standard deviation | | Offset mean | |
|  |  | Skewness | | Offset standard deviation | |
|  |  | Kurtosis | |  | |
|  |  | Uniformity | |  | |
|  |  | Entropy | |  | |

ISZ: intensity variance and size zone variance value; GLCM: gray-level co-occurrence matrix; NGTDM: Neighborhood gray tone difference matrix; LoG: Laplacian of Gaussian

*These features are calculated from whole, inner 2/3, and outer 1/3 ROI. Difference (delta) between inner and outer ROIs is computed.

**These features are calculated from the setting of * plus sub-sampled ROIs.

***The sigma value for LoG features were computed with σ = 0.5 – 3.5 in 0.5 increments.

Table S2. Definition of extracted radiomics features

|  | **Parameter** | **Formula** | **Description** | **IBSI** [2] **compliant** |
| --- | --- | --- | --- | --- |
| **Histogram-based features** [3] | Max, Min | $\mathrm{Max}=Max(X(i))$ or $\mathrm{Min}=Min(X(i))$  Where $X$ denotes the 3d image matrix with $N$ voxel. | Measures maximum or minimum intensity value of a histogram | O |
|  | Median | $Median=\frac{X(i)}{2}$  Where $X$ denote the 3d image matrix | Measures median intensity value of a histogram | O |
|  | Mean | $Mean=\frac{1}{N}\sum_{i}^{N} X(i)$  Where $X$ denote the 3d image matrix with $N$ voxel. | Measures mean intensity value of a histogram | O |
|  | Variance | $Variance=\frac{1}{N-1}\sum_{i=1}^{N} \left( X\left( i \right)-\bar{x} \right)^{2}$ | Measures squared distances of each value of a histogram from the mean | O |
|  | Standard deviation | $Std=\left( \frac{1}{N-1}\sum_{i=1}^{N} \left( X\left( i \right)-\bar{x} \right)^{2} \right)^{1/2}$  Where $X$ denote the 3d image matrix with $N$ voxel. | Measures amount of variation of a histogram. | O |
|  | Energy | $Energy=\sum_{i}^{N} {X(i)}^{2}$  Where $X$ denote the 3d image matrix with $N$ voxel. | Measures squared magnitude value of a histogram | O |
|  | Skewness | $Skewness=\frac{E{(x-\mu)}^{3}}{\sigma^{3}}$  Where $\mu$ is the mean of $x$, $\sigma$ is the standard deviation of $x$, $E$ is the expectation operator. | Measures asymmetry of a histogram. | O |
|  | Kurtosis | $Kurtosis=\frac{{E(x-\mu)}^{4}}{\sigma^{4}}$  Where $\mu$ is the mean of$x$, $\sigma$ is the standard deviation of $x$, $E$ is the expectation operator. | Measures “peakedness” of a histogram (flatness of histogram) | O |
|  | Root mean square (RMS) | $RMS=\sqrt{\frac{1}{N}\sum_{n=1}^{N} \left\vert X_{n} \right\vert^{2}}$  Where $X$ denote the 3d image matrix with $N$ voxel. | Measures the square-root of the mean of the squares of the values of the histogram. This feature is another measure of the magnitude of a histogram | O |
|  | Inter quartile range | $IQR=Q_{3}-Q_{1}$  Where $Q_{3}$ denote the 3^rd^ quartile of histogram, $Q_{1}$ denote the 1^st^ quartile of histogram | Measures of variability, based on dividing a histogram into quartiles | O |
|  | Range | $Range=range(X\left( i \right))$ | Measures difference between the highest and lowest voxel values of a histogram | O |
|  | Percentile | $Percentile=\left( \frac{n^{th} percentile}{100} \right) X(i)$ | Measures intensity value at the 2.5^th^, 25^th^ ,50^th^ ,75^th^, and 97.5^th^ percentile on histogram | O |
|  | Entropy | $\mathrm{Entropy}=-\sum_{i=1}^{N_{l}} P(i)\log_{2} P(i)$  Where $P$ denotes the first-order histogram with $N_{l}$ discrete intensity levels. | Measures irregularity of a histogram. | O |
|  | Uniformity | $Uniformity=\sum_{i=1}^{N_{l}} {P(i)}^{2}$  Where $P$ denotes the first-order histogram with $N_{l}$ discrete intensity levels. | Measures uniformity of a histogram. | O |
|  | Mean value of positive pixels (MPP) | $\mathrm{MPP}=\frac{1}{N_{+}}\sum_{i}^{N} X(i)$  Where $N_{+}$ denotes the total number of positive gray level pixels in $X\left( i \right)$ | Measures average positive histogram value. | X |
|  | Uniformity value of positive pixels (UPP) | $UPP=\sum_{i=1}^{N_{l}} \left\vert P(i) \right\vert^{2}$  Where $P$ denotes the first-order histogram with $N_{l}$ discrete intensity levels. | Measures uniformity of positive histogram value. | X |
| **Shape- and size-based features** [3,4] | Compactness | $Compactness=\frac{V}{\sqrt{\pi}A^{\frac{3}{2}}}$  Where$V$ denotes the volume, and $A$ denotes the surface area of the volume of interest (VOI) | Quantifies how close an object is to the smoothest shape, the circle | O |
|  | Surface area | $SA=\sum_{i=1}^{N} \frac{1}{2}\left\vert a_{i}b_{i}\times a_{i}c_{i} \right\vert$  Where $N$ is the total number of triangles (coved surface area), and $a,b, c$ are edge vectors | The surface area of the ROI | O |
|  | Convexity | $\mathrm{Convexity}=\frac{V}{V^{'}}$  Where $V$ denotes tumor volume, and $V^{'}$ denotes convex hull volume | Measures ratio of the ROI volume contained within the tumor to the calculated convex hull volume | O |
|  | Sphericity | $Sphericity=\frac{\pi^{\frac{1}{3}}\times{(6V)}^{\frac{2}{3}}}{A}$  Where $A$ denotes area, and $V$ denotes tumor volume | Measures of the roundness of the ROI | O |
|  | Spherical disproportion | $Spherical disproportion=\frac{A}{4\pi R^{2}}$  Where $R$ is the radius of a sphere with the same volume as the tumor | The ratio of the surface area of the ROI to the surface area of a sphere with the same volume as the ROI | O |
|  | Maximum 3D diameter | See description in the next column | Measures of the maximum 3D ROI diameter. It is measured as the largest pairwise Euclidean distance, between surface voxels of the ROI | O |
|  | Surface to volume ratio (SVR) | $SVR=\frac{A}{V}$  Where $A$ is area, and $V$ is volume | Surface to volume ratio in ROI | O |
|  | Volume | $Volume=R*number of voxels$  Where $R$ denote the 3d image resolution | Volume of tumor (ROI) | O |
|  | Mass | $Mass=V*D$  Where $V$ denote the tumor volume, $D$ denotes the tumor density | Mass of tumor (ROI) | O |
|  | Density | $Density=\frac{M}{V}$  Where $V$ denote the tumor volume, $M$ denote the tumor mass | Density of tumor (ROI) | O |
|  | Roundness factor (2D) | $Roundness factor=\frac{4\pi\cdot Area}{{Perimeter}^{2}}$ | Measure of circularity of a ROI | X |
|  | Eccentricity (2D) | $Eccentricity=c/a$  Where $c$ is the distance from the center to a focus and $a$ is the distance from that focus to a vertex | Measure of how the tumor shape is close to  the circle | X |
|  | Solidity (2D) | $Solidity= \frac{Area}{Convex area}$ | Measure of convexity of a ROI on the 2D image | X |
| **GLCM-based features** [3] | Auto correlation | $Autocorrelation =\sum_{i=1}^{N_{g}} \sum_{j=1}^{N_{g}} ij\boldsymbol{P}(i,j)$ | Measures of the magnitude of the fineness and coarseness of texture | O |
|  | Cluster tendency | $Cluster tendency=\sum_{i=1}^{N_{g}} \sum_{j=1}^{N_{g}} \left[ i+j-\mu_{x} -\mu_{y} \right]^{2}\mathbf{P}(i,j)$ | Measures of the homogeneity of GLCM | O |
|  | Maximum probability | $Maximum probability=max\{P\left( i,j \right)\}$ | Measures maximum value of GLCM matrix | X |
|  | Contrast | $Contrast=\sum_{i=1}^{N_{g}} \sum_{j=1}^{N_{g}} \left\vert i-j \right\vert^{2}\mathbf{P}(i,j)$ | Measures of the local intensity variation of GLCM | O |
|  | Difference entropy | $Difference entropy=\sum_{i=0}^{N_{g}-1} \mathbf{P}_{x-y}(i)\log_{2} [P_{x-y}(i)]$ | Measures entropy of processed GLCM matrix Px-y | O |
|  | Dissimilarity | $Dissimilarity=\sum_{i=1}^{N_{g}} \sum_{j=1}^{N_{g}} \left\vert i-j \right\vert\mathbf{P}(i,j)$ | Measures difference of each element of the gray level | O |
|  | Energy | $Energy=\sum_{i=1}^{N_{g}} \sum_{j=1}^{N_{g}} \left[ \mathbf{P}\left( i,j \right) \right]^{2}$ | Measures of the homogeneity of GLCM | O |
|  | Entropy | $Entropy=-\sum_{i=1}^{N_{g}} \sum_{j=1}^{N_{g}} \mathbf{P}(i,j)\log_{2} [\mathbf{P}\left( i,j \right)]$ | Measures irregularity of gray level. | O |
|  | Homogeneity | $Homogeneity=\sum_{i=1}^{N_{g}} \sum_{j=1}^{N_{g}} \frac{\mathbf{P}\left( i,j \right)}{1+\left\vert i-j \right\vert}$ | Measures closeness of gray-level. | O |
|  | Informational measure of correlation | $IMC=HXY-\frac{HXY1}{max\{HX,HY\}}$ | Secondary measure of Homogeneity | O |
|  | Variance | $Variance=\sum_{i=1}^{N_{g}} \sum_{j=1}^{N_{g}} \left( i-\mu_{x} \right)^{2}P(i,j)$ | Measures dispersion of the parameter values around the mean of the combinations of reference and neighborhood pixels | O |
|  | Where $\mathbf{P}\left( i,j \right)$is the gray level co-occurrence matrix for ($\delta=1, \alpha=0)$,  $N_{g}$is the number of discrete intensity value in the image,  $N$ is the number of voxels in the ROI,  $\mu$ is the mean of $\mathbf{P}\left( i,j \right),$  $p_{x}\left( i \right)=\sum_{j=1}^{N_{g}} \mathbf{P}(i,j)$ is the marginal row probabilities,  $p_{y}\left( i \right)=\sum_{i=1}^{N_{g}} \mathbf{P}(i,j)$ is the marginal column probabilities,  $\mu_{x}$ is the expected value of marginal row probability,  $\mu_{y}$ is the expected value of marginal column probability,  $\sigma_{x}$ is the standard deviation of $p_{x}$,  $\sigma_{y}$ is the standard deviation of $p_{y}$,  $p_{x+y}\left( k \right)=\sum_{i=1}^{N_{g}} \sum_{j=1}^{N_{g}} \mathbf{P}\left( i,j \right) , i+j=k, k=2,3,\ldots,2N_{g}$,  $p_{x-y}\left( k \right)=\sum_{i=1}^{N_{g}} \sum_{j=1}^{N_{g}} \mathbf{P}\left( i,j \right) ,\left\vert i-j \right\vert=k, k=0,1,\ldots,N_{g}-1$,  $HX=-\sum_{i=1}^{N_{g}} \mathbf{P}_{x}(i)\log_{2} \left[ p_{x}(i) \right]$ is the entropy of $\mathbf{P}_{x}$,  $HY=-\sum_{i=1}^{N_{g}} \mathbf{P}_{y}(i)\log_{2} \left[ p_{y}(i) \right]$ is the entropy of $\mathbf{P}_{y}$,  $HXY=-\sum_{i=1}^{N_{g}} \sum_{j=1}^{N_{g}} \mathbf{P}\left( i,j \right)\log_{2} \left[ \mathbf{P}(i,j) \right]$is the entropy of $\mathbf{P}\left( i,j \right)$  $HXY1=-\sum_{i=1}^{N_{g}} \sum_{j=1}^{N_{g}} \mathbf{P}\left( i,j \right)\log(p_{x}\left( i \right)p_{y}\left( j \right))$ | | | |
| **ISZ-based features** [5] | Size-zone variability | $Size zone variability=\frac{1}{\Theta}{\sum_{m=1}^{M} \left[ \sum_{n=1}^{N} \boldsymbol{P}\left( m,n \right) \right]}^{2}$ | Variability in the size of ROI | O |
|  | Intensity variability | $Intensity variability=\frac{1}{\Theta}{\sum_{n=1}^{N} \left[ \sum_{m=1}^{M} \boldsymbol{P}\left( m,n \right) \right]}^{2}$ | Variability in the intensity of ROI | O |
|  | Where $\boldsymbol{P}\left( m,n \right)$ is the intensity size zone matrix  $\Theta$ represents the number of homogeneous areas in tumor,  $M$ is the number of distinct intensity values,  $N$ is the size of homogeneous area in the matrix $\boldsymbol{P}\left( m,n \right)$ | | | |
| **NGTDM-based features** [6,7] | Busyness | $Busyness=\sum_{i=1}^{L} p_{i}s(i)/\sum_{i=1}^{L} \sum_{j=1}^{L} (ip_{i}-jp_{j})$ | Measure of spatial rate of gray-level change | O |
|  | Coarseness | $\mathrm{Coarseness}=\left[ \sum_{i=1}^{L} p_{i}s(i) \right]^{-1}$ | Measure of edge density | O |
|  | Complexity | $\mathrm{Complexity}=\sum_{i=1}^{L} \sum_{j=1}^{L} \left\{ \left( \left\vert i-j \right\vert\right)/(n^{2}\left( p_{i}+p_{j} \right)) \right\}\left\{ P_{i}s\left( i \right)+p_{j}s(j) \right\}$ | Measure of the amount of information in a ROI (gray-level intensities, number of sharp edges) | O |
|  | Contrast | $\mathrm{Contrast}=\frac{1}{N_{g}(N_{g}-1)}\sum_{i=1}^{L} \sum_{j=1}^{L} {p_{i}p_{j}\left( i-j \right)}^{2}\cdot\frac{1}{n^{2}}\sum_{i=1}^{L} s(i)$ | Measure of local variations and spread of matrix values | O |
|  | Strength | $\mathrm{Strength}=\sum_{i=1}^{L} \sum_{j=1}^{L} ({p_{i}+p_{j})\left( i-j \right)}^{2}/\sum_{i=1}^{L} s(i)$ |  | O |
|  | Where $p_{i}$ is the probability of occurrence of gray level value, $s(i)$ is the NGTDM, $N_{g}$ is the total number of different gray levels in the ROI, $L$ is the number of possible gray levels | | | |
| **Filter-based features** [4]  **(LoG)** | Mean | $Mean=\frac{1}{N}\sum_{i}^{N} G(i)$  Where $G$ denote the filtered 3d image matrix with $N$ voxel. | Measurement of mean of ROI image processed by LoG filter | X |
|  | Max | $\mathrm{Max}=Max(G(i))$  Where $G$ denotes the filtered 3d image matrix with $N$ voxel. | Measurement of max intensity value of ROI image processed by LoG filter | X |
|  | Min | $\mathrm{Min}=Min(G(i))$  Where $G$ denotes the filtered 3d image matrix with $N$ voxel. | Measurement of minimum intensity value of ROI image processed by LoG filter | X |
|  | Median | $Median=\frac{G(i)}{2}$  Where $G$ denote the filtered 3d image matrix | Measurement of median intensity value of ROI image processed by LoG filter | X |
|  | Standard deviation (Std) | $Std=\left( \frac{1}{N-1}\sum_{i=1}^{N} \left( G\left( i \right)-\bar{G} \right)^{2} \right)^{1/2}$  Where $G$ denote the filtered 3d image matrix with $N$ voxel. | Measurement of standard deviation of ROI image processed by LoG filter | X |
|  | Skewness | $Skewness=\frac{E{(G-\mu)}^{3}}{\sigma^{3}}$  Where $\mu$ is the mean of $G$, $\sigma$ is the standard deviation of $G$, $E$ is the expectation operator. | Measurement of skewness of ROI image processed by LoG filter | O |
|  | Kurtosis | $Kurtosis=\frac{{E(G-\mu)}^{4}}{\sigma^{4}}$  Where $\mu$ is the mean of$G$ $\sigma$ is the standard deviation of $G$, $E$ is the expectation operator. | Measurement of kurtosis of ROI image processed by LoG filter | O |
|  | Uniformity | $Uniformity=\sum_{i=1}^{N_{l}} {P(i)}^{2}$  Where $P$ denotes the first-order histogram with $N_{l}$ discrete intensity levels. | Measurement of uniformity of ROI image processed by LoG filter | O |
|  | Entropy | $\mathrm{Entropy}=-\sum_{i=1}^{N_{l}} P(i)\log_{2} P(i)$  Where $P$ denotes the first-order histogram with $N_{l}$ discrete intensity levels. | Measurement of entropy of ROI image processed by LoG filter | O |
|  | $G\left( x,y,z,\sigma\right)=I\left( x,y,z \right)*\frac{1}{{\sigma\left( \sqrt{2\pi} \right)}^{3}}e^{-\frac{x^{2}+y^{2}+z^{2}}{2\sigma^{2}}}$  $\sigma=0.5-3.5, 0.5 increments$, where I(x,y,z) is the image, and * denote convolution | | | |
| **Fractal-based features** [8,9] | Lacunarity (Box-counting method) | See description in the next column | Measure of the texture or distribution of gaps within an image | X |
|  | Dimension  (Box-counting method) | $Fractal dimension=\lim_{r\to0} \frac{\log(N_{r})}{\log(1/r)}$  Where $N_{r}$ is the number of voxels, and $r$ is the each of different side lengths | Fractal dimension quantifies morphological complexity and provides information on the self-similarity properties | X |
|  | Fractal signature dissimilarity  (Blanket method) | See description in the next column | Measure of tumor heterogeneity infromation | X |
| **Sigmoid function-based features** [4] | Amplitude mean | See description in the next column | Mean of the amplitude values of all sampling lines | X |
|  | Amplitude standard deviation |  | Standard deviation of the amplitue values of all sampling lines | X |
|  | Slope mean |  | Mean of the slope values of all sampling lines | X |
|  | Slope standard deviation |  | Standard deviation of the slope values of all sampling lines | X |
|  | Offset mean |  | Mean of the offset values of all sampling lines | X |
|  | Offset standard deviation |  | Standard deviation of the offset values of all sampling lines | X |
|  | $\mathrm{Sigmoid}\left( x \right)=\frac{A}{e^{\boldsymbol{B\cdot x}}+1}+C$  Where $A$ is the amplitude, $B$ is the slope of the curve, and $C$ is the offset of the curve | | | |

ISZ: intensity variance and size zone variance value; GLCM: gray-level co-occurrence matrix; NGTDM: Neighborhood gray tone difference matrix; LoG: Laplacian of Gaussian; ISBI: Image biomarker standardization initiative

**Contribution of each radiomics feature**

In experiment 1, we identified nine features to be reproducible in both voxel geometry settings. Under the original voxel geometry setting, the top three features explained most of the variance. The maximum (histogram-based) explained 51.35%, maximum 3D diameter (shape-based) explained 24.93%, and dissimilarity (GLCM-based) explained 12.55% of the total explained variance (Supplementary Table X). The top three important features using OOB observations were maximum 3D diameter, entropy (GLCM-based), and dissimilarity (GLCM-based) features. Under the isotropic geometry setting, maximum (histogram-based) explained 54.45%, maximum 3D diameter (shape-based) explained 28.7%, and dissimilarity (GLCM-based) explained 9.99% of the total variance (Supplementary Table X). The top three important features using OOB observations were maximum 3D diameter (shape-based), maximum (histogram-based), and entropy (GLCM-based) features. In general, the features that explained a larger portion of the variance had higher feature importance.

In experiment 2, we identified five features to be reproducible among different bin settings. Under the 32-bin setting, maximum (histogram-based) explained 51.83%, difference entropy (GLCM-based) explained 27.74%, and entropy (GLCM-based) explained 20.18% of the total explained variance. The top three important features using OBB observation were entropy (GLCM-based), difference entropy (GLCM-based), and maximum (histogram-based) features. Under the 64-bin setting, maximum (histogram-based) explained 51.86%, difference entropy (GLCM-based) explained 26.17%, and entropy (GLCM-based) explained 21.79%. The top three important features using OBB observation were entropy (GLCM-based), difference entropy (GLCM-based), and maximum (histogram-based) features. Under the 128-bin setting, maximum (histogram-based) explained 51.83%, entropy (GLCM-based) explained 24.56%, and difference entropy (GLCM-based) explained 23.12%. The top three important features using OBB observation were entropy (GLCM-based), difference entropy (GLCM-based), and homogeneity (GLCM-based) features. As in experiment 1, the features that described a larger portion of the variance had higher feature importance.

Table S3. The proportion of explained variance for reproducible features

| **Experiment** | **Name of the features** | **Explained variance [%]** |
| --- | --- | --- |
| **Experiment 1 – original voxel geometry** | maximum (histogram-based) | 51.35% |
|  | maximum 3D diameter (shape-based) | 24.93% |
|  | dissimilarity (GLCM-based) | 12.55% |
|  | Entropy (GLCM-based) | 10.5% |
|  | Cluster tendency  (GLCM-based) | 0.21% |
|  | Lacunarity (Fractal-based) | 0.14% |
|  | Skewness (Filter-based) | 0.14% |
|  | Minimum (Histogram-based) | 0.14% |
|  | Spherical disproportion  (shape-based) | 0.07% |
| **Experiment 1 – isotropic voxel geometry** | maximum (histogram-based) | 54.45% |
|  | maximum 3D diameter  (shape-based) | 28.7% |
|  | dissimilarity (GLCM-based) | 9.99% |
|  | Spherical disproportion  (shape-based) | 3.34% |
|  | Entropy (GLCM-based) | 2.26% |
|  | Skewness (Filter-based) | 1.17% |
|  | Cluster tendency  (GLCM-based) | 0.04% |
|  | Lacunarity (Fractal-based) | 0.04% |
|  | Minimum (Histogram-based) | 0% |
| **Experiment 2 – 32 bin** | maximum (histogram-based) | 51.83% |
|  | difference entropy  (GLCM-based) | 27.74% |
|  | entropy (GLCM-based) | 20.18% |
|  | Homogeneity (GLCM-based) | 0.13% |
|  | Minimum (Histogram-based) | 0.11% |
| **Experiment 2 – 64 bin** | maximum (histogram-based) | 51.86% |
|  | difference entropy  (GLCM-based) | 26.17% |
|  | entropy (GLCM-based) | 21.79% |
|  | Minimum (Histogram-based) | 0.11% |
|  | Homogeneity (GLCM-based) | 0.07% |
| **Experiment 2 – 128 bin** | maximum (histogram-based) | 51.83% |
|  | entropy (GLCM-based) | 24.56% |
|  | difference entropy  (GLCM-based) | 23.12% |
|  | Homogeneity (GLCM-based) | 0.37% |
|  | Minimum (Histogram-based) | 0.11% |


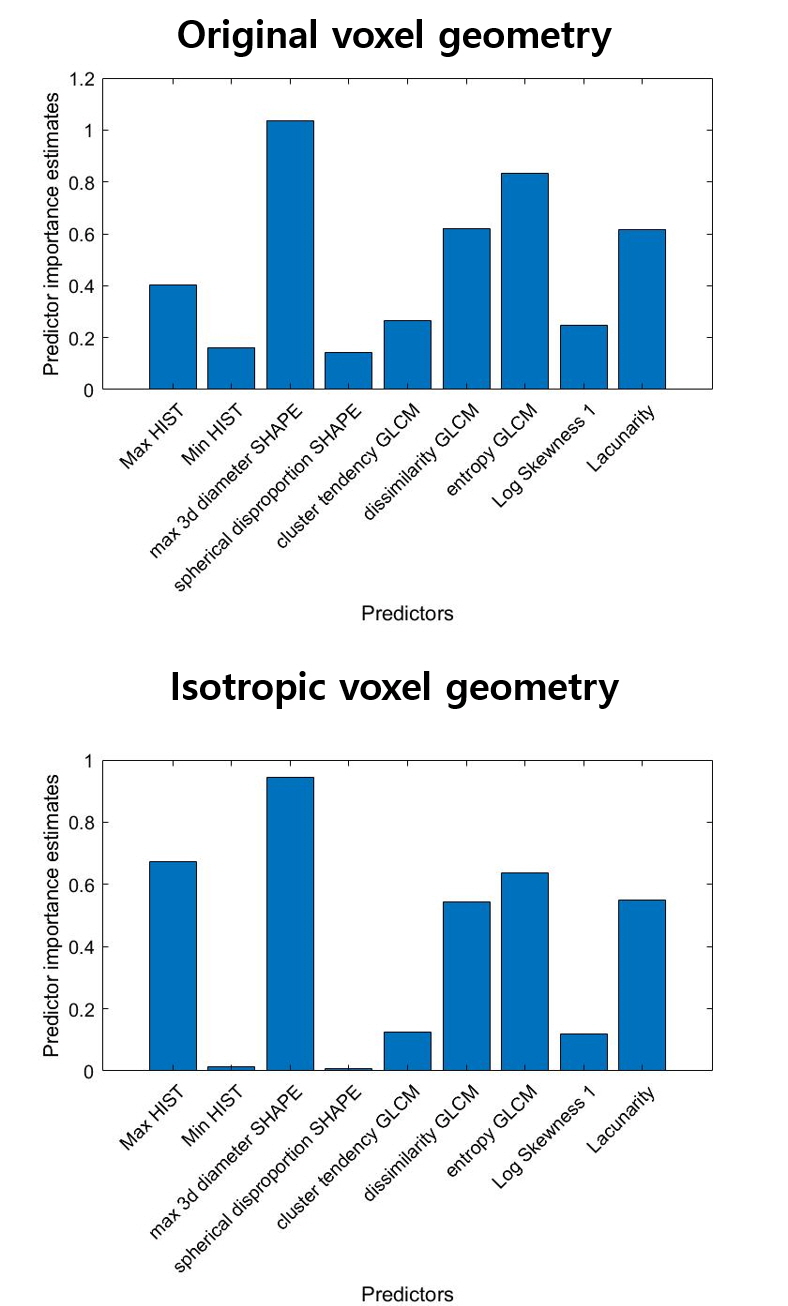


**Figure S1.** The relative importance of the features using permutation of out-of-bag (OOB) observations within the RF classifier in experiment 1. (A) original voxel geometry; (B) isotropic voxel geometry.


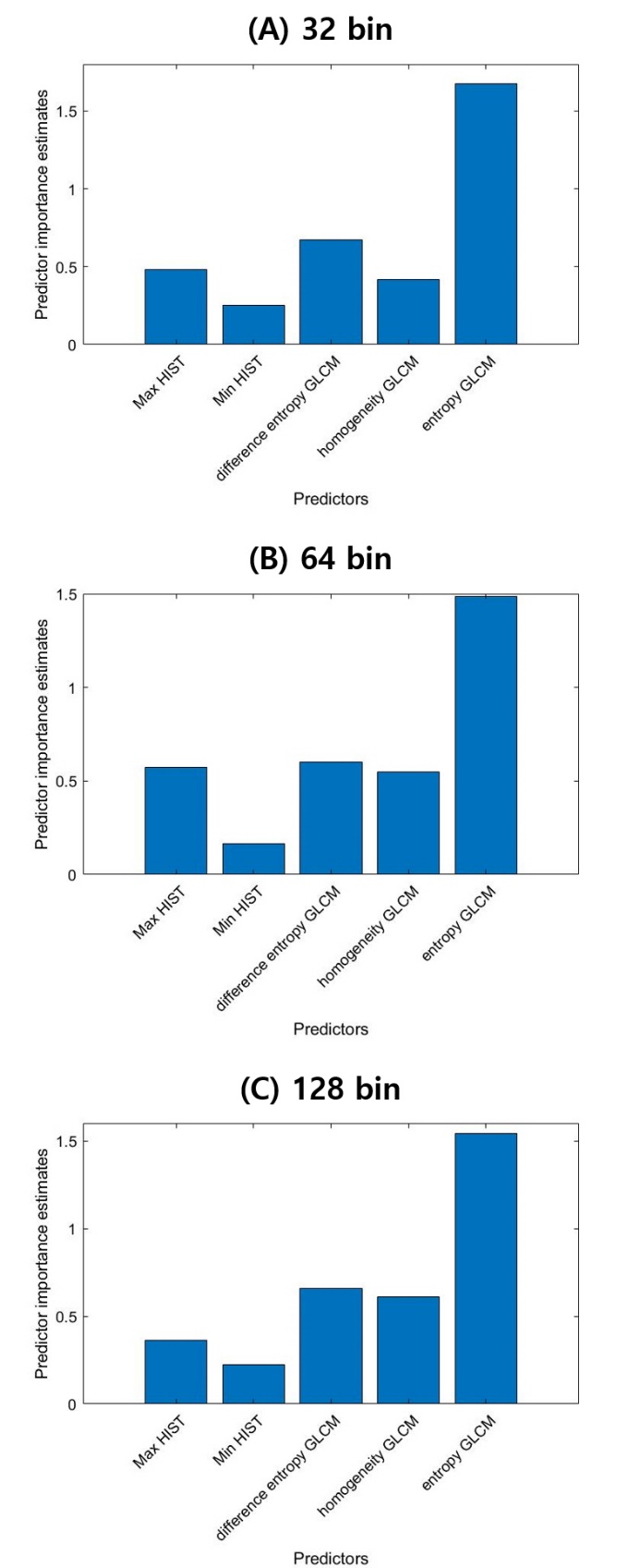


**Figure S2.** The relative importance of the features using permutation of out-of-bag (OOB) observations within the RF classifier in experiment 2. (A) 32 bin; (B) 64 bin; (C) 128 bin.**Supplementary Reference**

1. Van Griethuysen JJM, Fedorov A, Parmar C, Hosny A, Aucoin N, Narayan V, et al. Computational radiomics system to decode the radiographic phenotype. Cancer Res. 2017;77:e104–7.

2. Alex Zwanenburg, Stefan Leger, Martin Vallières SL. The image biomarker standardisation initiative. arXiv:1612.07003v9. 2016;

3. Aerts HJWJ, Velazquez ER, Leijenaar RT, Parmar C, Grossmann P, Carvalho S, et al. Decoding tumour phenotype by noninvasive imaging using a quantitative radiomics approach. Nat Commun. 2014;5:4006.

4. Aerts HJWL, Grossmann P, Tan Y, Oxnard GG, Rizvi N, Schwartz LH, et al. Defining a Radiomic Response Phenotype: A Pilot Study using targeted therapy in NSCLC. Sci Rep. Nature Publishing Group; 2016;6.

5. Chong Y, Kim JH, Lee HY, Ahn YC, Lee KS, Ahn MJ, et al. Quantitative CT variables enabling response prediction in neoadjuvant therapy with EGFR-TKIs: Are they different from those in neoadjuvant concurrent chemoradiotherapy? PLoS One. 2014;9:1–8.

6. Niu L, Qian M, Yang W, Meng L, Xiao Y, Wong KKL, et al. Surface Roughness Detection of Arteries via Texture Analysis of Ultrasound Images for Early Diagnosis of Atherosclerosis. PLoS One. 2013;8.

7. Davnall F, Yip CSP, Ljungqvist G, Selmi M, Ng F, Sanghera B, et al. Assessment of tumor heterogeneity: An emerging imaging tool for clinical practice? Insights Imaging. 2012;3:573–89.

8. Northshore GCC, Lennon FE, Cianci GC, Cipriani N a, Hensing T a, Zhang HJ, et al. Lung cancer—a fractal viewpoint. Nat Publ Gr. Nature Publishing Group; 2015;12:664–75.

9. Wang C, Subashi E, Yin FF, Chang Z. Dynamic fractal signature dissimilarity analysis for therapeutic response assessment using dynamic contrast-enhanced MRI. Med Phys. 2016;43:1335–47.
